# Activated *Luffa* derived biowaste carbon for enhanced desalination performance in brackish water†

**DOI:** 10.1039/c9ra01872g

**Published:** 2019-05-14

**Authors:** Deepa Sriramulu, Sareh Vafakhah, Hui Ying Yang

**Affiliations:** Pillar of Engineering Product Development, Singapore University of Technology and Design Singapore 487372 yanghuiying@sutd.edu.sg

## Abstract

Membrane capacitive deionization (MCDI) is an effective process to remove salt ions from brackish water. In this work, a systematic investigation was carried out to study the effects of applied potential and salt concentration on salt adsorption capacity (SAC), charge efficiency (*Λ*) and energy consumption in an MCDI system using *Luffa* biowaste derived carbon as electrodes. We studied the comparative MCDI performance of *Luffa* derived carbon as electrodes before and after activation. Furthermore, the desalination capacities of the electrodes were quantified by batch-mode experiments in a 2500 mg L^−1^ NaCl solution at 0.8–1.2 V. Activated *Luffa* carbon showed a high SAC of 38 mg g^−1^ at 1.2 V in a 2500 mg L^−1^ NaCl solution with a low energy consumption of 132 kJ mol^−1^ salt as compared to non-activated samples (22 mg g^−1^, 143 kJ mol^−1^). The adsorption mechanisms were investigated using kinetic models and isotherms under various applied potentials. Consequently, the excellent SAC of activated *Luffa* carbon can be attributed to the presence of micro/mesoporous network structure formed due to the activation process for the propagation of the salt ions.

## Abstract 2

Membrane capacitive deionization (MCDI) is an effective process to remove salt ions from brackish water.

## Graphical abstract

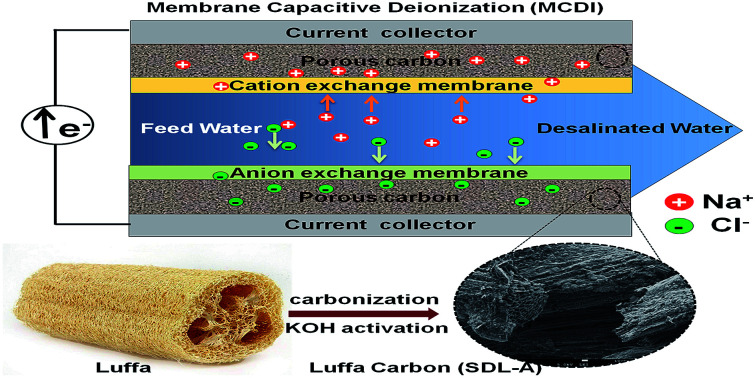

## 1 Introduction

Currently, we are facing a surge in global demand for freshwater and this has put pressure on our limited water supplies.^1,2^ In response, many countries have adopted desalination as a means to supplement water supplies. Technologies such as reverse osmosis, thermal distillation and electrodialysis have all been thoroughly developed to effectively desalinate water at an industrial scale.^3–5^ Despite this, the high costs of production, energy requirements and hazardous by-products prevent these technologies from being fully sustainable.^6,7^ As such, there is a need to investigate alternative methods of desalination for an environmentally viable solution.

One of the most promising solutions is capacitive deionization (CDI). In a typical CDI process, an external potential is imposed across two electrodes as water is allowed to pass through a channel between them. Charged species in water are extracted and stored within electrical double layers (EDLs) at the surfaces of electrodes while de-ionized water is produced. A short-circuit is then applied to regenerate the electrodes for the next adsorption phase.^8,9^ CDI can potentially rival conventional desalination methods due to low energy costs, simple infrastructure and long operational lifetimes.^1,2,6^ However, industrial adoption of CDI is hampered by low salt adsorption capacity (SAC) of its electrode materials and low charge efficiency of operation. To alleviate these problems, ion-exchange membranes are placed in front of electrodes in a format called membrane capacitive deionization (MCDI).^10^ An anion-exchange membrane placed in front of the anode forces the anode to only accept or reject anions whereas a cation-exchange membrane placed in front of the cathode does the opposite. This prevents the expulsion of co-ions during electrode charging^11^ and improves overall SAC and charge efficiency. Furthermore, a reverse potential can be used to regenerate the electrodes. This is not possible in traditional CDI since ions would be re-adsorbed onto the opposing electrode.

To complement the advantages of MCDI, much research has been devoted to developing electrode materials with high surface area, better pore size distribution and superior electrical conductivity.^12^ Among them, carbon aerogels,^13,14^ graphene composites,^15–17^ carbon nanotubes,^18,19^ nanofibers,^20,21^ mesoporous carbons,^22,23^ activated carbons,^24,25^ have been studied as promising electrode materials for MCDI. Unfortunately, most of these materials were synthesized using petroleum-derived chemicals, which are limited and non-renewable involving tedious synthetic process thus enhancing the cost of production applications.^26^ Therefore, developing low-cost, eco-friendly carbon-based electrode materials for high-performance CDI is of high importance.

Biowaste carbons refer to porous carbon materials derived from naturally biodegradable waste products. These carbon materials usually inherit the unique porous structures of their biowaste parents and have been shown to be effective for CDI.^27–29^ For instance, watermelon peel derived carbon^28^ was able to deliver a SAC of about 17.38 mg g^−1^ at 1.2 V in a 500 mg L^−1^ NaCl solution while pomelo melon derived carbon delivered a SAC 20.78 mg g^−1^ at 1.4 V in a 1000 mg L^−1^ NaCl solution.^27^*Luffa* was also used as biowaste for carbon and exhibited a SAC of 20 mg g^−1^ in a 500 mg L^−1^ NaCl solution at 1.0 V using an MCDI system. However, the said work did not adequately study the effects of potential and salt concentration on electrosorption performance. Furthermore, the work did not provide energy consumption results by the system during adsorption.^30^

Herein, a systematic investigation was carried out to identify the effects of applied potential and salt concentration on SAC, charge efficiency (*Λ*) and energy consumption (kJ mol^−1^ salt removed) in an MCDI system using *Luffa* derived biowaste carbon. We compared the electrosorption performances of *Luffa* derived carbon before (SDL-C) and after chemical activation using KOH^31^ (SDL-A). Activated materials are known to possess high surface areas suitable for ion adsorption and functional groups on activated surfaces can improve wettability. Indeed, our experiments show better electrochemical and deionization performances for SDL-A over SDL-C. Low energy consumption was also recorded for SDL-A when brackish salinities were used. Our experimental results highlight the feasibility of using biowaste carbons such as *Luffa* as electrodes materials for desalination of brackish water.

## 2 Experimental

### 2.1 Materials

*Luffa* sponge was sourced from commercial markets. Potassium hydroxide (KOH) and concentrated hydrochloric acid (HCl, 36%) were purchased from Sigma-Aldrich. All chemicals were of AR grade and used without further purification.

### 2.2 Preparation of *Luffa* derived biowaste carbon (SDL-C and SDL-A)

In a typical synthesis, *Luffa* sponge was cut into pieces and carbonized under Ar atmosphere at 800 °C for 1 h. This sample was denoted as SDL-C. To activate SDL-C, SDL-C and KOH were first mixed in a mass ratio of 1 : 2 in 30 mL deionized water for 10 h then dried in an oven at 80 °C. The mixture was then heated to 800 °C at a rate of 5 °C min^−1^ and kept at for 800 °C 1 h. The product was finally cooled to room temperature before it was washed thoroughly with 1 M HCl to remove any inorganic salts. The sample was washed for a second time with deionized water to remove the acid and dried in an oven at 80 °C overnight. The activated sample was denoted as SDL-A.

### 2.3 Physical and chemical characterization

The morphology of the carbonized samples was observed by field-emission scanning electron microscopy (FESEM, JEOL JSM-7600F). Powder X-ray diffraction (XRD) patterns were recorded using a Bruker D8 Advance diffractometer with Cu Kα (*λ* = 0.154 nm) radiation at 40 kV. Nitrogen adsorption–desorption isotherms were recorded at 77 K using a Quantachrome Autosorb-IQ gas sorption analyzer. The resulting specific surface area and pore size distribution were determined using multipoint Brunauer–Emmett–Teller (BET) and Barrett–Joyner–Halenda (BJH) methods. Raman spectra were obtained using a WITec confocal Raman system with 532 nm laser excitation. X-ray photoelectron spectroscopy (XPS) analysis was performed using PHI-5400 equipment with Al Kα beam source (250 W) and position-sensitive detector (PSD).

### 2.4 Electrochemical characterization

The capacitive performance of *Luffa* derived carbon was studied using cyclic voltammetry (CV) and electrochemical impedance spectroscopy (EIS) in a three-electrode configuration in a 1 M NaCl solution. The three-electrode system consists of a working electrode, a Pt foil as a counter electrode and a standard calomel electrode (SCE) as a reference. The working electrode was prepared by first mixing a slurry of 80 wt% active material, 10 wt% carbon black and 10 wt% polyvinylidene fluoride (PVDF) with *N*-methyl-2-pyrrolidone (NMP) as a solvent. This slurry was then coated onto a graphite current collector and dried at 80 °C overnight to form the working electrode (1 × 1 cm^2^, ∼1.2 mg). The specific capacitance (*C*, F g^−1^) was calculated using CV curves obtained from eqn (1)^32^ as follows:1
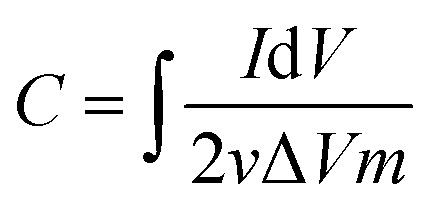
where *I* is the current density (A), Δ*V* is the potential window, *v* is the scan rate (V s^−1^) and *m* is the mass of the active material (g). All electrochemical experiments were conducted using a Bio-logic VMP3 electrochemical workstation.

### 2.5 Electrosorption experiments

**Fig. 1:**
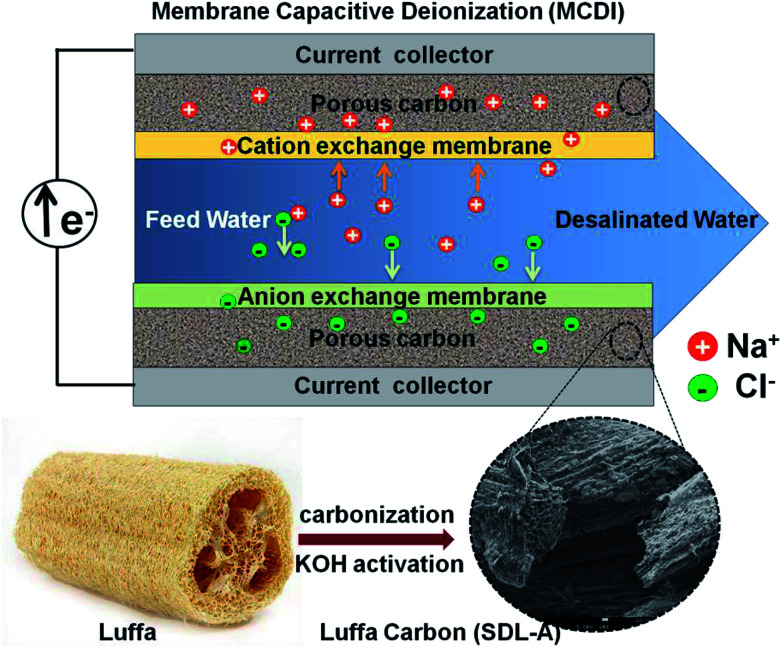
Schematic illustration of the preparation process of SDL-A from *Luffa* sponge for MCDI application.

The MCDI cell was constructed as shown in the schematic of Fig. 1. A photograph of the cell and the experimental setup is also provided in Fig. S1.† A typical MCDI setup consists of a pair of ion-exchange membranes (Hangzhou Iontech Environmental Technology Co. Ltd, China) placed in front of *Luffa* derived carbon electrodes sandwiching a nylon spacer. Electrodes were prepared in the same way as the ones used for electrochemical characterization but were of 4 × 4 cm^2^ and 200 μm thick. The total mass of active material, carbon black and PVDF was around 100 mg.

Batch mode experiments were conducted to evaluate the desalination performance of *Luffa* derived carbon electrodes. A NaCl solution (∼50 mL) was circulated between the MCDI cell and a reservoir tank using a peristaltic pump (BT100S, Lead Fluid) at a flow rate of 50 mL min^−1^. The electrical conductivity was monitored every 10 s at the outlet using a conductivity meter (DDSJ-308F, Leici). Electrosorption experiments were conducted by either varying the potential from 0.8 to 1.2 V at increments of 0.2 V or by changing the NaCl concentration from 500 to 2500 mg L^−1^ at increments of 500 mg L^−1^. The SAC (*Γ*, mg g^−1^), specific charge stored during the adsorption (*Σ*, C g^−1^) and charge efficiency (*Λ*) were calculated according to eqn (2), (3) and (4), respectively:^33^2
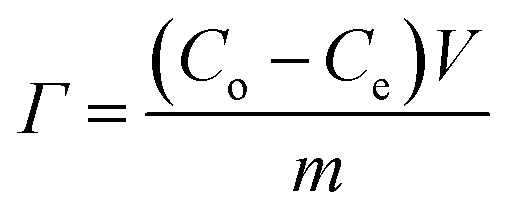
3
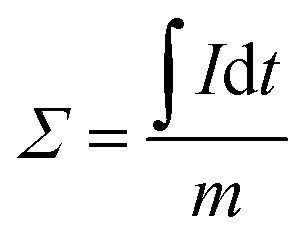
4
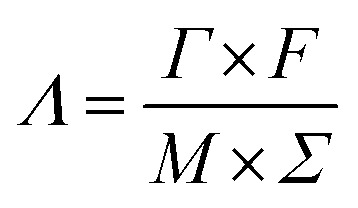
where *C*_o_ is the initial NaCl concentration (mg L^−1^), *C*_e_ is the final equilibrated NaCl concentration (mg L^−1^), *V* is the volume of NaCl solution (L), and *m* refers to the total mass of the two electrodes (g), *I* refers to the current during the adsorption process (A), *F* is the Faraday's constant (96 500 C mol^−1^) and *M* is the molar mass of NaCl (58.5 g mol^−1^). Another metric, the salt adsorption rate (SAR) is calculated according to the eqn (5).5
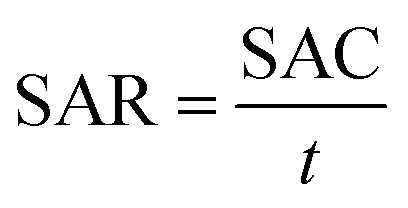


## 3 Results and discussion

### 3.1 Synthesis, structural and morphological characterization of *Luffa* derived carbon (SDL-A, SDL-C)

**Fig. 2:**
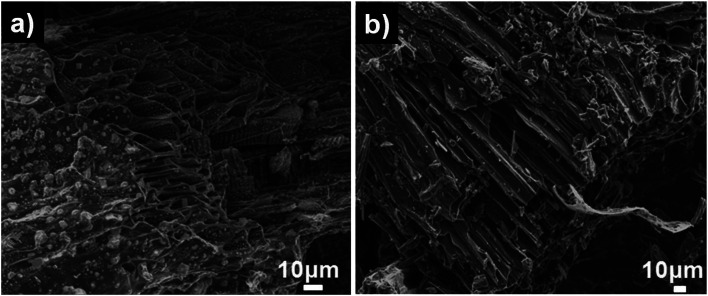
SEM images of (a) SDL-C and (b) SDL-A *Luffa* carbon.

Pristine *Luffa* sponge consists of an interconnected network of fibers^34^ (Fig. 1). After carbonization and KOH activation, a large porous structure was observed in SEM images of SDL-C and SDL-A (Fig. 2) due to the inherent fibrous structure of the *Luffa* sponge. These porous well-interconnected structures can serve as efficient pathways for rapid diffusion of ion species.

**Fig. 3:**
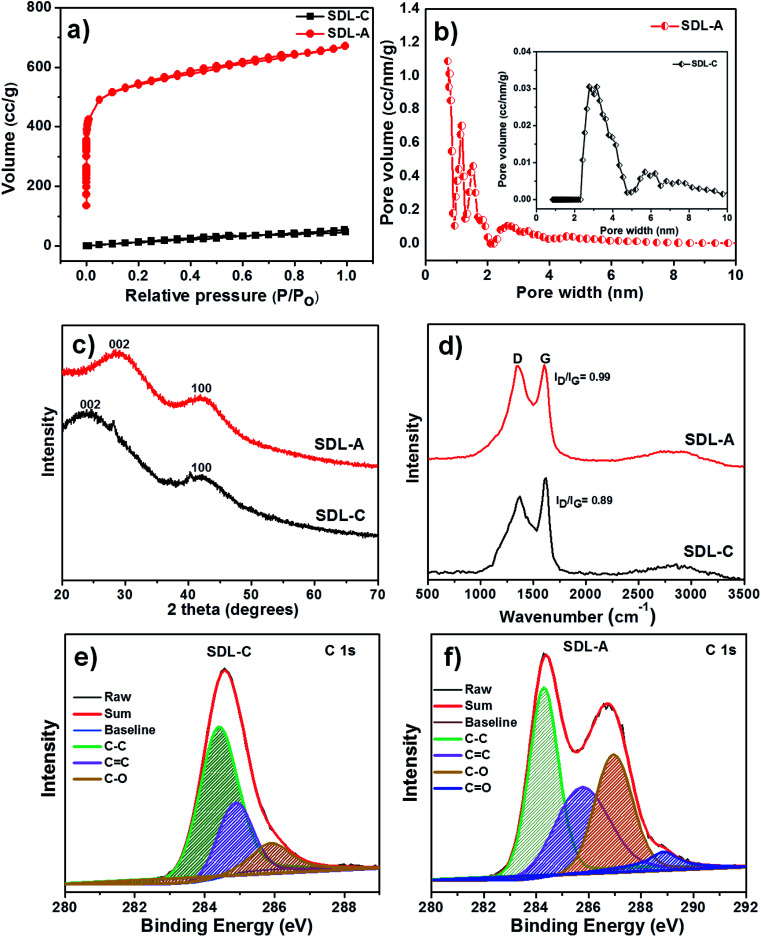
(a) Nitrogen adsorption–desorption isotherms, (b) pore size distribution (inset is the pore size distribution of SDL-C), (c) XRD pattern, (d) Raman spectra, (e) and (f) are C 1s XPS spectra of SDL-C and SDL-A materials.

The surface area and pore structure of SDL carbon before and after activation were further investigated using N_2_ adsorption–desorption curves as shown in Fig. 3a. SDL-C exhibited a very low nitrogen uptake and the BET surface area was only 69 m^2^ g^−1^ while the pore size distribution ranged between 2–10 nm which implied the presence of mesopores. On the contrary, SDL-A exhibited a high surface area of 2062 m^2^ g^−1^ and a broad pore size distribution ranging from 0.5–4 nm. N_2_ isotherm of SDL-A was a combination of type I and type II isotherms and featured an increase in N_2_ adsorption at *P*/*P*_o_ < 0.05 caused by capillary filling of micropores and a wide knee at higher pressure *P*/*P*_o_ > 0.05 which implied the existence of narrow mesopores (Fig. 3b). Judging from the high surface area and hierarchical pore size distribution, we expect SDL-A to provide more active sites for the adsorption of ions.^35,36^

The crystallographic structure was determined based on powder XRD patterns and Raman spectra (Fig. 3c and d). From Fig. 3c, we observed two broad diffraction peaks representing (002) and (100) reflections of amorphous graphitic carbon in SDL-C and SDL-A.^37^ There was a shift in (002) peak after activation from 2*θ* = 23.8° to 28.5° which indicated a decrease in the interlayer distance (*d*_002_) and an increase in crystallinity. Fig. 3d shows the Raman spectra of SDL-C and SDL-A. Both samples exhibited bands at 1357 cm^−1^ (D band) and 1601 cm^−1^ (G band). D band corresponds to defects and disorders in graphitic structures whereas G band refers to in-plane vibrations of sp^2^ bonded carbon structures.^38^ Further, the intensity ratio (*I*_D_/*I*_G_) of D and G bands was used to estimate the graphitization degree of carbon. *I*_D_/*I*_G_ peak ratios were 0.98 for SDL-A and 0.89 for SDL-C which suggested a greater degree of disorder and defects in SDL-A. This result can be attributed to the highly porous structure of SDL-A.

XPS measurements were employed to investigate the chemical composition of SDL-C and SDL-A carbon. As shown in Fig. S2a,† the full spectrum of both SDL-C and SDL-A exhibited two peaks at 284.8, and 351.9 eV corresponding to C 1s and O 1s respectively. High-resolution C 1s XPS spectrum was also obtained (Fig. 3e and f) and can be deconvoluted into four peaks at a binding energies of 284.4, 285.7, 286.6 and 288.6 eV corresponding to C–C, C=C, C–O and C=O respectively.^23,39^ Upon activation with KOH and carbonization, the carbon content SDL-A increased as compared to SDL-C samples. Furthermore, the high resolution O 1s spectrum shown in Fig. S2† contained three deconvoluted peaks at 531.5 eV (C=O), 532.9 eV (C–O) and 533.6 eV (C–O–C) respectively for both SDL-C and SDL-A.^40^ Accordingly, the presence of higher oxygen-containing groups on the surface of SDL-A electrodes enhances the wettability of the electrodes and consequently improves electrochemical performance.^41^

### 3.2 Electrochemical performance

**Fig. 4:**
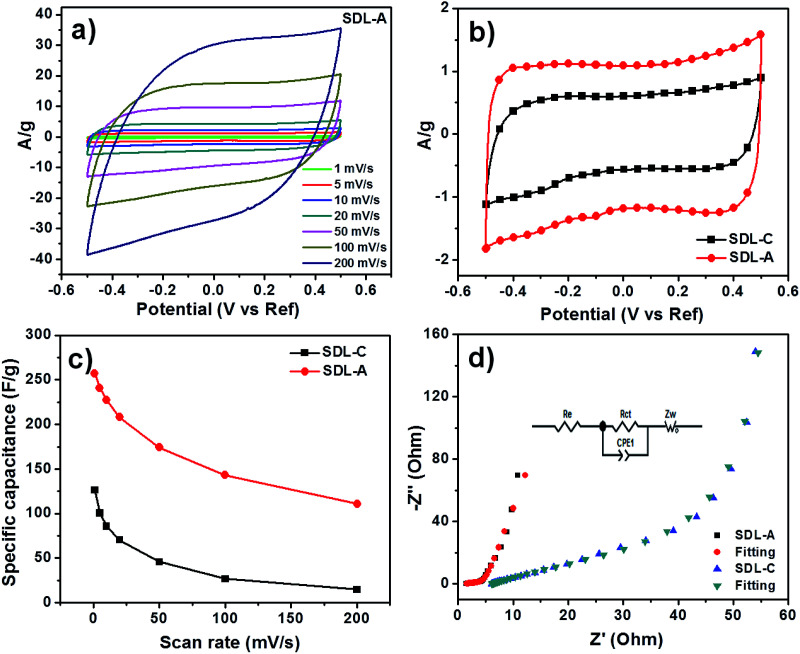
Electrochemical performance measured in a three-electrode system with 1 M NaCl electrolyte solution. (a) CV curves of SDL-A at a different scan rate from 1 mV to 200 mV s^−1^, (b) CV curves of SDL-C and SDL-A at a scan rate of 5 mV s^−1^, (c) specific capacitance of SDL-C and SDL-A at different scan rates and (d) Nyquist profiles of the electrodes under the influence of an ac voltage of 5 mV, inset are the electrical equivalent circuit used for fitting the impedance spectra of SDL-C and SDL-A.

Quasi-rectangular CV curves were observed for SDL-C and SDL-A at different scan rates (Fig. 4a and S3†) which implied a non-ideal capacitive behavior. We also observed a larger CV loop for SDL-A which meant it possessed a larger capacitance than SDL-C (Fig. 4b). A summary of specific capacitances is presented in Fig. 4c and the highest capacitance obtained by SDL-A was 270 F g^−1^ as opposed to SDL-C at 125 F g^−1^. This high capacitance could be attributed to factors such as high surface area, hierarchical pore size distribution and surface wettability of SDL-A. For both samples, a decrease in specific capacitance corresponding to an increase in scan rate was observed due to insufficient time for the electrolyte to diffuse into the inner pores of the electrode material.

Results of our EIS experiments are presented in Fig. 4d. The Nyquist plots of SDL-A and SDL-C showed a small semicircle at the high-frequency region which corresponded to the charge transfer resistance (*R*_ct_) at the electrode and electrolyte interface while a vertical line observed at the low-frequency region could be attributed to ion diffusion resistance. The parameters of the electronic elements in an equivalent circuit are shown in Table S1.† The fitted *R*_ct_ values of SDL-C and SDL-A were 61.9 and 4.1 Ω respectively. The smaller *R*_ct_ value of SDL-A shows how faster charge transfer can be achieved at the electrode and electrolyte interface due to a highly connected porous structure. At the low-frequency region, a straight line with the steepest slope was observed for SDL-A which implied a low Warburg impedance (*Z*_w_) and hence, the highest diffusion coefficients. SDL-A also showed the smallest *x*-intercept which implied the lowest internal resistance (*R*_e_). Thus, we expect SDL-A to possess the best electrosorption performance.

### 3.3 Desalination performance

**Fig. 5:**
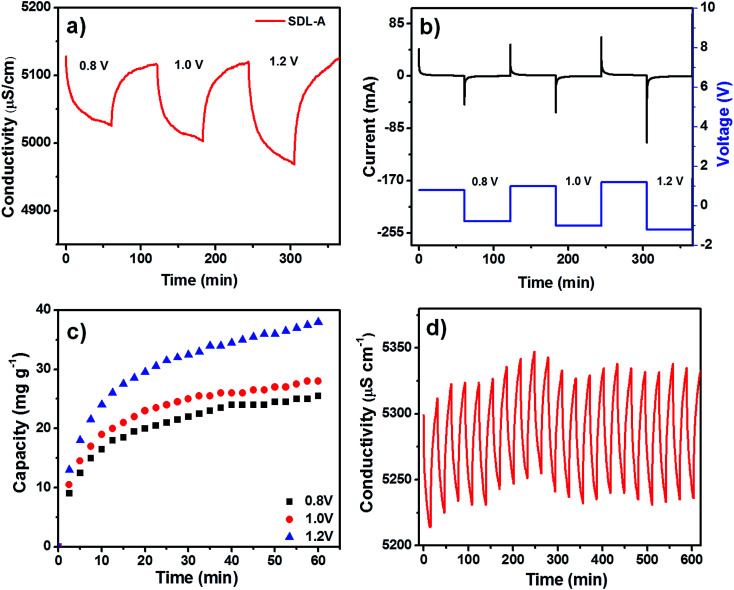
(a) Electrosorption behaviours of the SDL-A electrode at different voltages in NaCl solution with an initial concentration of ∼2500 mg L^−1^ with a flow rate of 50 mL min^−1^, (b) its corresponding current response, (c) salt removal capacity of SDL-A electrode at different voltages ranging from 0.8 V to 1.4 V in NaCl solution with an initial concentration of ∼2500 mg L^−1^ and, (d) deionization and regeneration curves of SDL-A electrodes in a 2500 mg L^−1^ NaCl solution at an applied voltage of 1.2 V.

A positive electrical potential is applied to the MCDI across the two electrodes during the charging step to adsorb ions from a solution of known NaCl concentration and a negative potential was applied to discharge the electrodes and expel the ions in a waste stream. Fig. 5a shows the desalination performance of SDL-A at an initial concentration of 2500 mg L^−1^ at various electrical potentials from 0.8 V to 1.2 V in NaCl solution. In all applied potentials, a sharp decrease in solution conductivity was first observed during charging and as adsorption approaches equilibrium, the change in conductivity decreases until a plateau is reached. Fig. 5b shows a positively correlated current transient with increasing potential while Fig. 5c shows the SAC of SDL-A electrode at different applied potentials. From 0.8 V to 1.2 V, the SAC increased from 23 to 38 mg g^−1^, indicating that a higher cell potential can enhance electrosorption capacity. The observed phenomenon can be attributed to the presence of strong electrostatic forces and the formation of a thicker EDL at an increased voltage. Fig. 5d shows the cyclic stability of SDL-A electrode in 2500 mg mL^−1^ NaCl solution over 20 charge–discharge cycles without any apparent loss in removal capacity. This result further confirmed that the SDL-A electrode can be fully regenerated and reused without any declination, making it a promising choice of electrode material for MCDI applications.

**Fig. 6:**
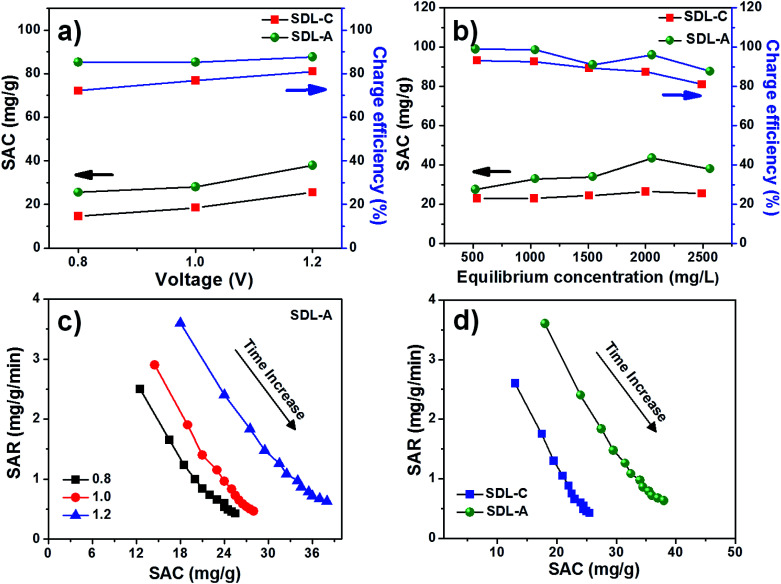
Comparison of SAC and charge efficiency of SDL-C and SDL-A electrodes (a) at different applied voltages 0.8–1.2 V, (b) at different initial concentrations of NaCl solutions at an applied voltage of 1.2 V, Ragone plots of SAR *vs.* SAC for (c) SDL-A electrode at different applied voltages in 2500 mg L^−1^ NaCl solution and (d) for SDL-C and SDL-A electrodes in 2500 mg L^−1^ NaCl solution at an applied voltage of 1.2 V with a flow rate of 50 mL min^−1^.

Fig. 6a shows the electrosorption capacity and charge efficiency related to SDL-C and SDL-A. At each applied voltage, the removal capacity and corresponding charge efficiency of SDL-A were higher than SDL-C electrodes. The charge efficiency increased gradually from 83 to 87% with an increase in applied voltage from 0.8 to 1.4 V for SDL-A and 70 to 80% for SDL-C electrodes. This observation is consistent with reports in literature.^42,43^ The highest SAC for SDL-A was at 38 mg g^−1^ at a charge efficiency of 80% when the applied potential was 1.2 V. The MCDI performance was further evaluated different initial NaCl concentrations ranging from 500 to 2500 mg L^−1^ at an applied voltage of 1.2 V as shown in Fig. 6b. SAC gradually increases with an increase in the initial concentration with SDL-A electrode exhibiting higher SAC as compared to SDL-C. For instance, at a concentration of 500 mg L^−1^, the SAC of SDL-A is 38 mg g^−1^ and 22 mg g^−1^ for SDL-C electrodes. With an increase in saline concentration, electrosorption capacity increases due to the enhancement of ion diffusion inside the pores and a reduction of EDL overlapping effects.^44^ A Ragone plot was also used to understand the deionization performance of the electrodes in our MCDI system.^45^Fig. 6c shows the Ragone plot of SAR *vs.* SAC of the SDL-A electrode under different applied potentials. The performance of SDL-A electrode at an applied potential of 1.2 V shifted towards the upper right region, indicating that SAR and SAC increase at higher applied potentials. The observed increase in SAC and SAR at high potentials was due to an increase in coulombic interaction between the electrode and charged ions, which increases the thickness of EDL and thus enhancing the desalination performance at high voltage.^46^ Seen in Fig. 6d is the Ragone plot for SDL-C and SDL-A electrodes in 2500 mg L^−1^ NaCl aqueous solution at an applied potential of 1.2 V. The performance of SDL-A is marked at the upper right corner which indicates an increase in SAC and SAR as compared to the SDL-C electrode. This observation could be attributed to an increase in surface area and development of a hierarchical micro/mesoporous structure due to the activation of carbon.

We had also studied the adsorption kinetics of the adsorption process.^47,48^ Pseudo-first-order and pseudo-second-order adsorption kinetics equations as shown below were used for our analyses.

Pseudo-first-order

6 *q*_*t*_ = *q*_e_(1 − exp^−*k*_1_*t*^)

Pseudo-second-order7
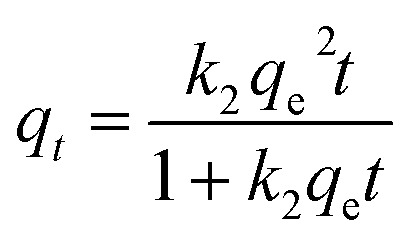
where *q*_e_ (mg g^−1^) and *q*_*t*_ (mg g^−1^) are the amounts of ions adsorbed at equilibrium and at a certain time *t* (min) respectively. *k*_1_ (mg g^−1^ min^−1^) and *k*_2_ (g mg^−1^ min^−1^) are the adsorption rate constants of pseudo-first-order and pseudo-second-order equations respectively. Fitting between experimental data and the equations are as shown in Fig. S4a.† The values of the coefficients (*q*_e_, *k*_1_, *k*_2_) were calculated from the slopes and intercept values of the plots in Fig. S4a† and summarised in Table 1. Pseudo-second order kinetics models fit the experimental data the best with regression coefficients for second order kinetics >0.99.

**Table 1:** Coefficient of pseudo-first and second-order kinetic models

Kinetic models and parameters	0.8 V	1.0 V	1.2 V
*q*_e_ (exp) (mg g^−1^)	25.5	28	38

**Pseudo-first-order**			
*q*_e_ (cal) (mg g^−1^)	23.7	26.1	35.15
*k*_1_ (min^−1^)	0.1180	0.1327	0.1120
*R* ^2^	0.9336	0.9415	0.9427

**Pseudo-second-order**			
*q*_e_ (cal) (mg g^−1^)	27.2	29.6	40
*k*_2_ (g mg^−1^ min^−1^)	0.0053	0.0056	0.0033
*R* ^2^	0.9847	0.9904	0.9876

Adsorption isotherms were also used to predict the electrosorption behaviour and predict the maximum SAC of SDL-A electrodes in NaCl solution.^48^ The Langmuir isotherm was used to fit the experimental data and equations are as shown as follows:8
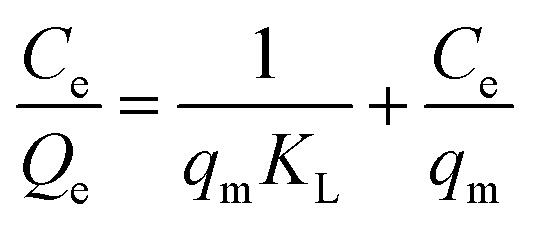
where *Q*_e_ (mg g^−1^) is the amount of NaCl adsorbed per gram of adsorbent, *C*_e_ (mg L^−1^) is the equilibrium concentration, *q*_m_ (mg g^−1^) is the maximum adsorption capacity corresponding to complete monolayer adsorption, and *K*_L_ (L mg^−1^) refers to the Langmuir constant. Fig. S4b† shows the electrosorption isotherm of SDL-A electrode at a cell potential of 1.2 V and different initial concentrations ranging from 500–2500 mg L^−1^ NaCl solution. The plot of specific adsorption 
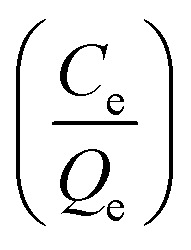
 against the equilibrium concentration shows a good correlation with the Langmuir model. The Langmuir parameters and the regression coefficients were determined from the slope and the intercepts are summarized in ESI Table S2.† Accordingly, *q*_m_ (45 mg g^−1^) of SDL-A calculated from the Langmuir isotherm equation was higher than that of SDL-A electrode (38 mg g^−1^), implying an enhanced electrosorption behaviour.

**Fig. 7:**
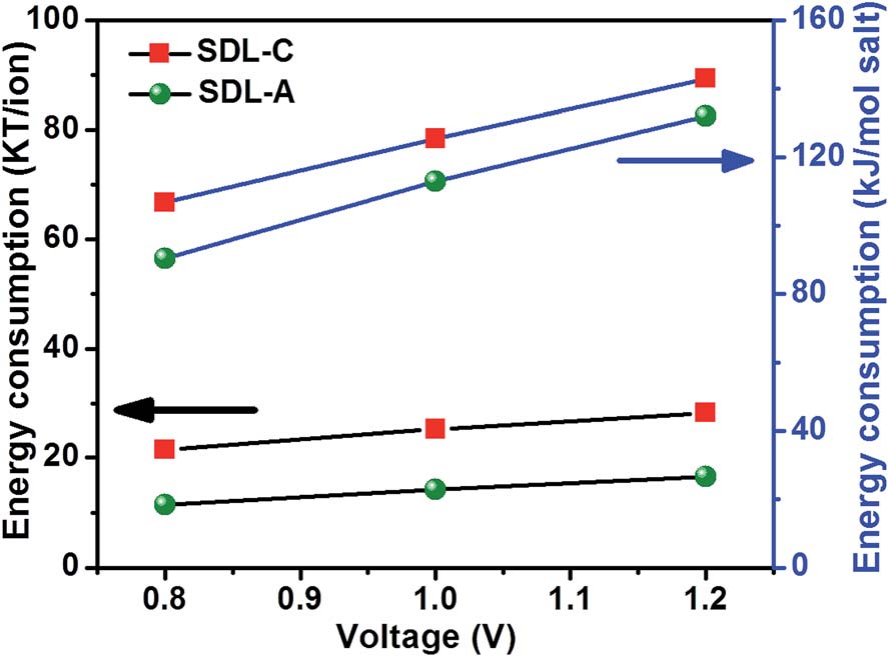
Effect of applied voltage on MCDI energy consumption using SDL-C and SDL-A electrodes at an initial concentration of 2500 mg L^−1^ NaCl aqueous solution with a flow rate of 40 mL min^−1^.

The energy consumed by SDL-C and SDL-A electrode to remove an amount of salt from saline water at an applied potential is shown in Fig. 7. The specific energy consumption (kJ mol^−1^ of salt removed) was calculated using eqn (9) below:9
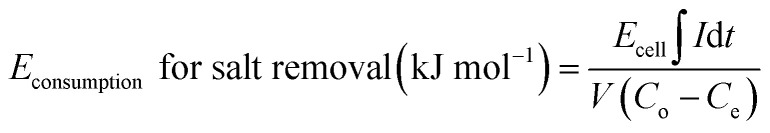
where *E*_cell_ (V) is the potential applied to the MCDI system and all other parameters were as defined previously. Energy required to remove an ion (kT per ion) was calculated based on a literature reported elsewhere.^11^ As observed in Fig. 7, specific energy consumption increased with an increase in the applied potential for both SDL-A and SDL-C electrodes, which is in accordance with previous reported work. The energy consumption obtained for *Luffa* derived carbons was around 18–28 kT, which is comparable with values reported for commercial MCDI.^11^ Even though the energy consumption of commercial MCDI is comparable with our MCDI system using biocarbon as electrodes. The advantage of biocarbon electrode is the low cost and eco friendly process. The low cost of biowaste materials refers to easily available in abundance, and are easily recyclable. Further they can be efficiently converted to green carbon electrodes by physical or chemical activation. Thus, SDL-A electrode possessed a larger SAC (38 mg g^−1^) at 1.2 V in a NaCl concentration of 2500 mg L^−1^ with lower energy consumption (132 kJ mol^−1^) as compared to SDL-C electrodes (22 mg g^−1^, 142 kJ mol^−1^). Part of this low energy consumption was attributable to the inclusion of ion exchange membranes.

## 4 Conclusions

In summary, *Luffa* derived carbons were successfully prepared using *Luffa* sponges. The prepared carbon materials exhibited a micro/mesoporous hierarchical structure with a high specific surface area of 2062 m^2^ g^−1^ and excellent electrochemical properties. They were developed as highly efficient electrodes for MCDI to remove salt from brackish water. We managed to achieve a high SAC of 38 mg g^−1^ in a 2500 mg L^−1^ of NaCl solutions at 1.2 V for SDL-A electrodes, which was 72% higher than that of SDL-C electrode (22 mg g^−1^). A combination of meso- and micropores in SDL-A electrode decreases the resistance of NaCl ions when diffusing through the porous carbon and micropores provide a large surface area for ion adsorption. Our results show efficient KOH activated electrodes can be developed for MCDI desalination using low cost biowaste products.

## Conflicts of interest

The authors declare no competing financial interest.

## Supplementary Material

RA-009-C9RA01872G-s001
