# Supplementary material for: Activated Luffa derived biowaste carbon for enhanced desalination performance in brackish water
Source: RSC Adv. 2019 May 14;9(26):14884–92. doi: 10.1039/c9ra01872g (PMC9064238; doi:10.1039/c9ra01872g)
Supplement: RA-009-C9RA01872G-s001 [file RA-009-C9RA01872G-s001.pdf]

Electronic Supplementary Material (ESI) for RSC Advances.
This journal is © The Royal Society of Chemistry 2019

Supporting Information

Activated Luffa derived biowaste carbon for enhanced desalination performance in brackish water

Deepa Sriramulu, Sareh Vafakhah and Hui Ying Yang*

Pillar of Engineering Product Development,

Singapore University of Technology and Design, Singapore 487372

yanghuiying@sutd.edu.sg

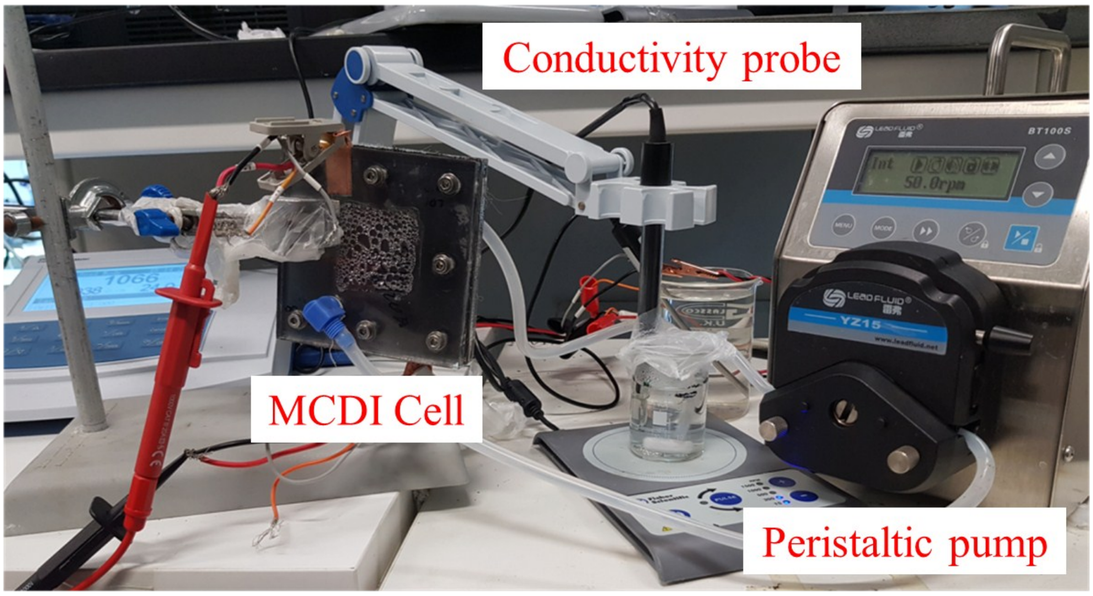

Fig. S1 MCDI experimental setup.

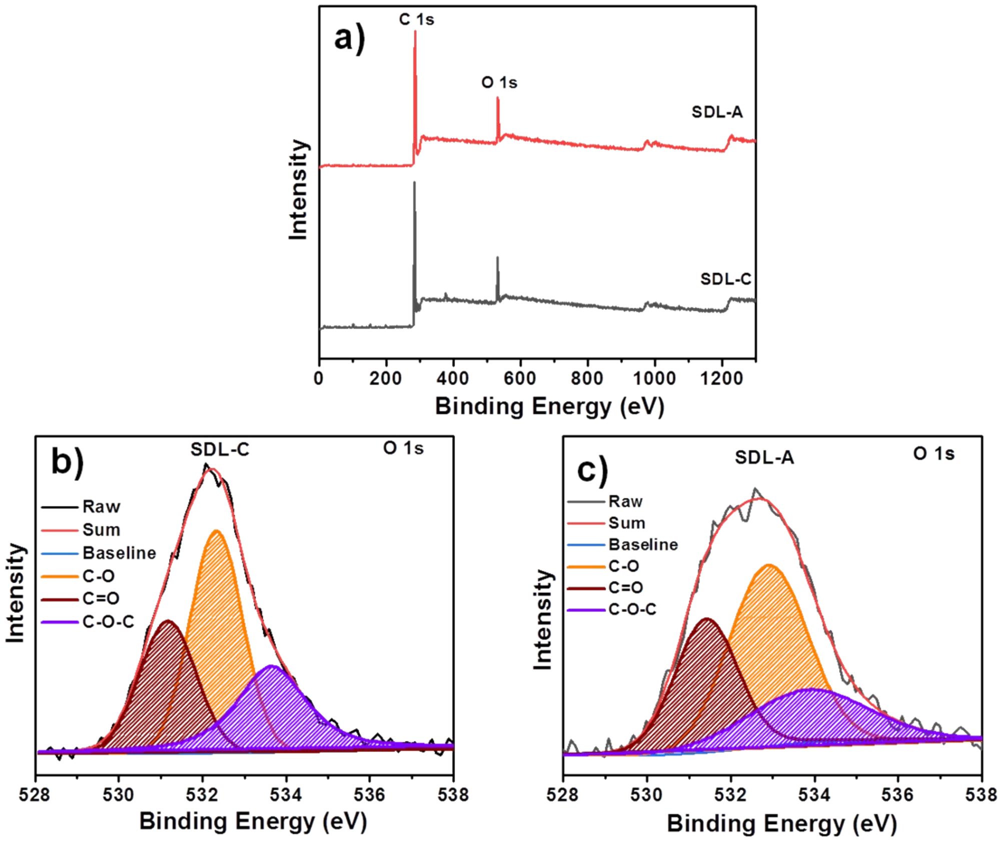

Fig. S2 (a) Full scan XPS spectrum of SDL-C and SDL-A and High resolution spectra of O 1s for (b) SDL-C and (c) SDL-A.

Table. S1 Parameters of electronics elements in the equivalent circuit in Figure 4a

**Sample**	**R_e (Ω)**	**R_{ct} (Ω)**	**Z_w (Ω)**
SDL-C	6.001	61.9	1.976
SDL-A	1.457	4.129	0.086

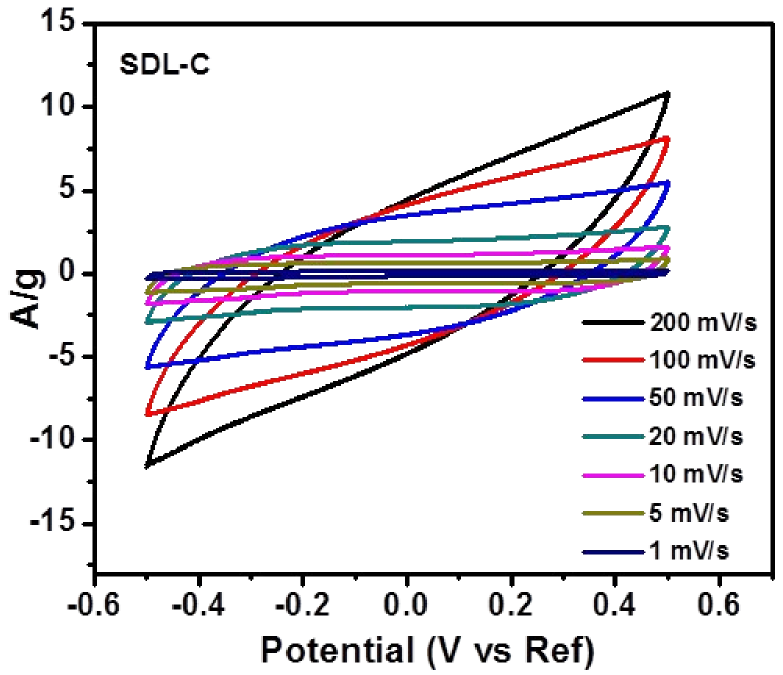

Fig. S3 CV curves of SDL-C at a different scan rate from 1 mV to 200 mV/s, measured in a three-electrode system with 1M NaCl electrolyte solution.

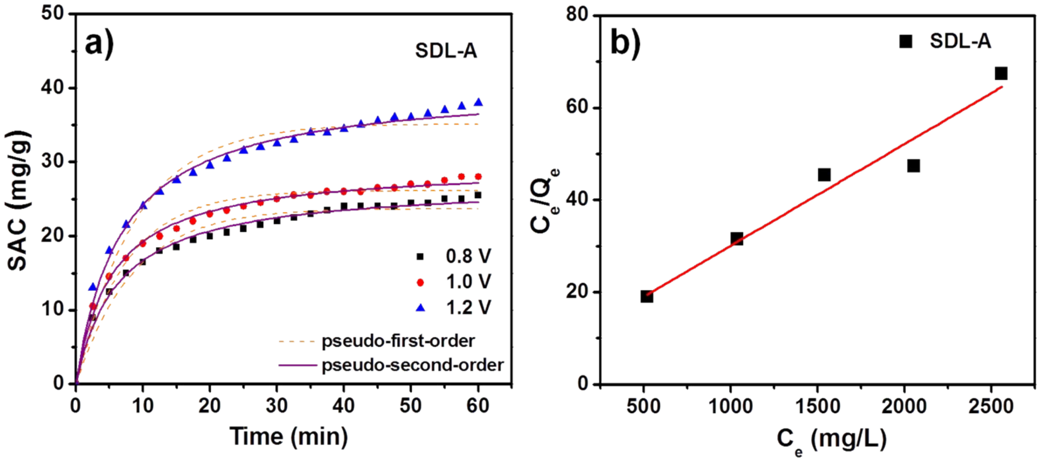

Fig. S4. (a) The electrosorption kinetics of NaCl onto the SDL-A electrodes at different voltages, and (b) Langmuir adsorption isotherm of NaCl onto the SDL-A at different equilibrium concentrations at 1.2 V.

Table S2 Parameters of Langmuir isotherm study of SDL-A in 2500 mg/L NaCl solution at 1.2 V

**Sample**	**R_e (Ω)**
Linear equation	Y= 0.02209x+ 7.98835
Coefficients	q_m= 45 mg/g,
	K_L= 0.0027 L/mg
R^2	0.94234
